# Hyperlocal Variation in Soil Iron and the Rhizosphere Bacterial Community Determines Dollar Spot Development in Amenity Turfgrass

**DOI:** 10.1128/AEM.00149-21

**Published:** 2021-04-27

**Authors:** Ming-Yi Chou, Smita Shrestha, Renee Rioux, Paul Koch

**Affiliations:** a Department of Plant Pathology, University of Wisconsin–Madison, Madison, Wisconsin, USA; Nanjing Agricultural University

**Keywords:** disease variation, dollar spot, microbiome, pathogen suppression, phytobiome, plant-soil-microbe interaction, rhizosphere, soil iron, soil microbiology, turfgrass

## Abstract

Dollar spot, caused by the fungal pathogen *Clarireedia* spp., is an economically important foliar disease of amenity turfgrass in temperate climates worldwide. This disease often occurs in a highly variable manner, even on a local scale with relatively uniform environmental conditions. The objective of this study was to investigate mechanisms behind this local variation, focusing on contributions of the soil and rhizosphere microbiome. Turfgrass, rhizosphere, and bulk soil samples were collected from within a 256-m^2^ area of healthy turfgrass, transported to a controlled environment chamber, and inoculated with Clarireedia jacksonii. Bacterial communities were profiled by targeting the 16S rRNA gene, and 16 different soil chemical properties were assessed. Despite their initial uniform appearance, the samples differentiated into highly susceptible and moderately susceptible groups following inoculation in the controlled environment chamber. The highly susceptible samples harbored a unique rhizosphere microbiome with suggestively lower relative abundance of putative antibiotic-producing bacterial taxa and higher predicted abundance of genes associated with xenobiotic biodegradation pathways. In addition, stepwise regression revealed that bulk soil iron content was the only significant soil characteristic that positively regressed with decreased dollar spot susceptibility during the peak disease development stage. These findings suggest that localized variation in soil iron induces the plant to select for a particular rhizosphere microbiome that alters the disease outcome. More broadly, further research in this area may indicate how plot-scale variability in soil properties can drive variable plant disease development through alterations in the rhizosphere microbiome.

**IMPORTANCE** Dollar spot is the most economically important disease of amenity turfgrass, and more fungicides are applied targeting dollar spot than any other turfgrass disease. Dollar spot symptoms are small (3 to 5 cm), circular patches that develop in a highly variable manner within plot scale even under seemingly uniform conditions. The mechanism behind this variable development is unknown. This study observed that differences in dollar spot development over a 256-m^2^ area were associated with differences in bulk soil iron concentration and correlated with a particular rhizosphere microbiome. These findings provide interesting avenues for future research to further characterize the mechanisms behind the highly variable development of dollar spot, which may inform innovative control strategies. Additionally, these results suggest that small changes in soil properties can alter plant activity and hence the plant-associated microbial community, which has important implications for a broad array of agricultural and horticultural plant pathosystems.

## Abstract (precis)

Dollar spot is the most economically important disease of amenity turfgrass, and more fungicides are applied targeting dollar spot than any other turfgrass disease. Dollar spot symptoms are small (3 to 5 cm), circular patches that develop in a highly variable manner within plot scale even under seemingly uniform conditions.

## INTRODUCTION

Dollar spot is a foliar pathogen of cool-season turfgrasses in North America that is caused by the fungus *Clarireedia* spp. and is the most economically important disease of amenity turfgrass in the world (1). It causes roughly circular patches of bleached turfgrass 3 to 5 cm in diameter that can blight the stand and reduce the functionality of the site for recreational purposes (2). The primary host of dollar spot is creeping bentgrass (Agrostis stolonifera), and a lack of host resistance or effective cultural control strategies has made dollar spot the target of more fungicide applications than any other turfgrass disease (3). Heavy reliance on synthetic fungicides has led to the development of fungicide-resistant fungal populations (4), imposes a significant financial burden on the turfgrass manager (5), and increases the risk of human and environmental contamination resulting from repeated chemical exposures (6). The development of dollar spot symptoms in uniformly managed turfgrass with nearly identical environmental conditions is often highly variable, even at a scale of several meters (7). It is unclear why dollar spot develops in such a variable manner, but plausible explanations include a link to hyperlocal variations in microbial antagonists or local variations in soil physical, chemical, or biological properties.

Spatial variation in plant disease is often observed in both managed and natural plant systems, although most studies on variation in plant disease incidence and severity have been conducted in large-scale agricultural fields over tens or hundreds of hectares. Adiobo et al. (8) observed that the physicochemical and microbial properties of andosols suppressed Pythium myriotylum root rot in cocoyam (Xanthosoma sagittifolium) more effectively than ferralsols. Varied susceptibility to disease in adjacent fields with similar soil physicochemical characteristics has commonly been attributed to disease suppressive or disease conducive soils and is often influenced by cropping history (9, 10). Although on a larger scale than the variation observed in dollar spot, the pathogen suppression function of a specific suppressive soil has provided some clues as to how the same soil type could have dramatically different pathogen suppression functions. Specific disease suppressive soils and the associated antagonistic microbes have been found in various major cropping systems against important plant pathogens, such as Fusarium oxysporum, Phytophthora megasperma, and Gaeumannomyces graminis (11–13). Enrichment of an antagonistic microbial population in the rhizosphere often serves as the key plant pathogen suppression mechanism in previously characterized disease suppressive soils (14). As a classic example, enrichment of antibiotic 2,4-diacetylphloroglucinol-producing fluorescent *Pseudomonas* species in the rhizosphere of both wheat and flax has been shown to reduce take-all disease in both plants (15).

The rhizosphere microbiome and its functions are codetermined by both the plant and the soil. The host plant produces root exudates that recruit particular microbes from within the soil (14). The soil harbors varied microbial communities shaped by soil type and associated properties, such as physical structure and pH (16). Therefore, the rhizosphere microbiome and its microbial disease suppressive function can shift following changes in the soil environment. Peng et al. (17) varied the chemical and physical properties of soils found to be both suppressive and conducive to Fusarium oxysporum f. sp. *cubense* and demonstrated that soil physicochemical traits can mediate suppressiveness against the pathogen’s chlamydospores. This finding suggests that soil physicochemical and microbial properties can cooperatively improve plant disease suppression in agricultural fields.

Soil spatial variation in microbial properties is often studied at multiple levels, including micro, plot, field, landscape, and regional scales (18, 19). Over a small plot scale, spatial variation of smut disease (Ustilago syntherismae) on crabgrass (Digitaria sanguinalis) was influenced by both pathogen spore density and spatial location (20). However, soil property influences were not investigated in this study, and spores or other long-distance dispersal mechanisms have never been observed with dollar spot in a field environment (2). High spatial variations in soil physicochemical and microbial properties were observed in a managed grassland, including a wide range of soil pH, nitrogen content, microbial biomass, and microbial catabolism profiles within the scales of several centimeters to meters (21), but the impacts of these variations on plant-pathogen interactions were not studied. Recently, Wei et al. (22) examined disease variation in tomato (Solanum lycopersicum) and observed that the initial rhizosphere soil bacterial community could effectively predict the severity of the disease caused by the soilborne bacterial pathogen Ralstonia solanacearum. Similarly, in a field scale, Chen et al. (23) observed differences in rhizosphere bacterial community structure, diversity, acid phosphatase activity, root iron content, and bulk soil calcium and magnesium between healthy and poorly growing highbush blueberry plants (Vaccinium corymbosum) in an unidentified pathosystem. These results again indicate the importance of both soil chemical properties and the rhizosphere microbiome on plant health over a field scale or smaller. However, it remains unknown whether the rhizosphere and/or bulk soil microbiome impacts the severity of disease caused by a foliar fungal pathogen when interacting with specific soil chemical properties.

In this study, various factors contributing to the localized variation in dollar spot development on monocultured turfgrass were studied. Rhizosphere and bulk soil bacterial communities as well as bulk soil chemical properties were examined to determine possible causes for the highly variable spatial nature of dollar spot development. We hypothesized that soil chemical properties and the rhizosphere bacteria are both significant variables for determining dollar spot disease susceptibility in a uniformly managed and monocultured turfgrass system. Turfgrass is an excellent system in which to study this phenomenon because the high plant density allows for robust sampling over a small scale. The initial 132-cm^2^ surface area turfgrass soil plug harbored an estimated 1,200 individual creeping bentgrass plants, and each subsample derived from the soil core contained 10 to 15 individual plants. By understanding the factors that drive variation in dollar spot disease development within a plot scale in a high-density monoculture system, we may discover mechanisms that can be targeted for improved biological management of a number of important plant pathogens.

## RESULTS

### Dollar spot development.

**FIG 1:**
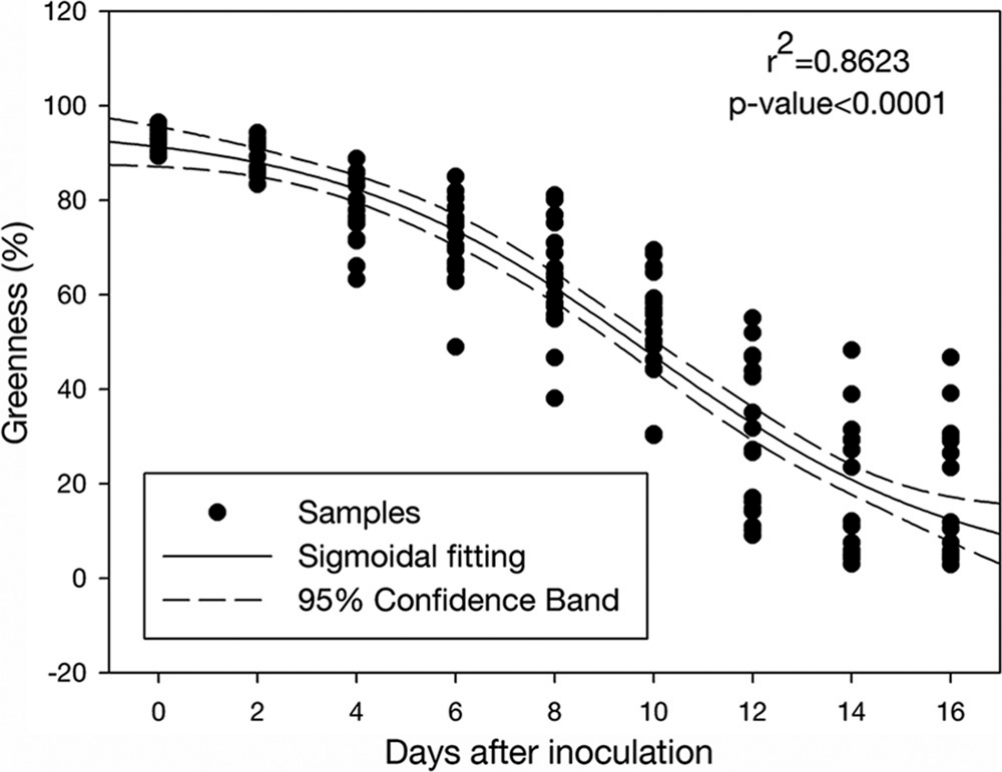
Dollar spot (*C. jacksonii*) development as indicated by turf greenness decay curve fitted with sigmoidal model (*r*^2^ = 0.8623, *P* < 0.0001) throughout 16 days of incubation after dollar spot inoculation (*n* = 18).

Dollar spot development was measured as a decrease of greenness over time in order to standardize the quantification of disease symptoms, as lesion shape and color can be difficult to determine with simple visual assessments. The resulting greenness decay curve followed a sigmoidal decay pattern (*r*^2^ = 0.8623 and *P* < 0.0001) (Fig. 1). Disease symptoms initially developed within 2 days after inoculation (DAI) and then increased rapidly over the next 4 to 12 DAI, before slowing during the saturation phase at 14 to 16 DAI. Substantial differences in symptom severity between samples started showing up 4 DAI, and differences remained apparent throughout the incubation.

### Attributing soil bacterial community difference as a function of disease variability.

Turf samples were grouped into high, medium, and low disease according to the disease severity at each DAI with a preplanned number of six samples in each group. The bacterial microbiome from rhizosphere and bulk soil associated with each sample, which had been separated prior to inoculation, was then assessed to see if the baseline microbiome structure explained turfgrass responses to Clarireedia jacksonii inoculation. The rhizosphere bacterial community differed between high and low disease severity groups when categorized based on severity between 4 and 10 DAI according to the permutational analysis of variance (PERMANOVA) (Table 1). There were no differences in bacterial community structure found between high and low disease severity groups when categorized according to initial disease development (DAI 0 to 2) or the disease saturation phase (DAI 12 to 16). In addition, no differences in the bulk soil bacterial community were found among the disease severity groups throughout the incubation (Table 1). The period where the rhizosphere soil bacterial community showed structural differences between the high and low disease groups (4 to 10 DAI) matched the backslope of the disease development curve (Fig. 1), which suggested that the initial soil rhizosphere bacterial community can affect the peak dollar spot development. The samples were then recategorized according to their disease status during the peak disease development stage (4 to 10 DAI) to make the peak disease development period the target of prediction instead of any single day within this period. The samples initially categorized as high disease during the period 4 to 10 DAI never shifted into the low severity group and vice versa, so the 18 samples naturally broke into 2 groups except for 1 sample that stayed in the medium disease group throughout the study and was excluded from further analysis. Further analyses were performed based on classifying the samples into nine highly susceptible (HS) samples and eight moderately susceptible (MS) samples.

**TABLE 1:** Paired PERMANOVA analysis of turf-associated soil microbiome prior to *C. jacksonii* inoculation^*a*^

Sample type	Pair comparison	0 DAI		2 DAI		4 DAI		6 DAI		8 DAI		10 DAI		12 DAI		14 DAI		16 DAI	
		*r*^2^	*P* value	*r*^2^	*P* value	*r*^2^	*P* value	*r*^2^	*P* value	*r*^2^	*P* value	*r*^2^	*P* value	*r*^2^	*P* value	*r*^2^	*P* value	*r*^2^	*P* value
Rhizosphere	High vs low	0.116	0.126	0.124	0.090	0.138	0.006**	0.139	0.003**	0.118	0.021*	0.126	0.024*	0.091	1.000	0.091	1.000	0.091	1.000
	High vs med	0.080	1.000	0.100	0.603	0.136	0.012*	0.112	0.090	0.103	0.345	0.128	0.036*	0.080	1.000	0.096	0.840	0.096	0.816
	Low vs med	0.115	0.117	0.089	1.000	0.103	0.147	0.116	0.036*	0.096	0.807	0.109	0.096	0.092	1.000	0.100	0.540	0.100	0.573
Bulk	High vs low	0.125	0.144	0.101	0.597	0.087	1.000	0.089	1.000	0.085	1.000	0.080	1.000	0.080	1.000	0.091	1.000	0.091	1.000
	High vs med	0.094	0.978	0.096	0.939	0.086	1.000	0.112	0.372	0.122	0.207	0.081	1.000	0.088	1.000	0.092	1.000	0.092	1.000
	Low vs med	0.093	0.984	0.087	1.000	0.076	1.000	0.088	1.000	0.099	0.549	0.080	1.000	0.076	1.000	0.068	1.000	0.068	1.000

a Categorized based on disease level (high, medium, and low) after inoculation of *C. jacksonii* and throughout the incubation. Asterix indicates the significance level: *, *P* < 0.05 and **, *P* < 0.01. DAI, days after inoculation of *C. jacksonii*.

### Comparison of rhizosphere bacterial communities of highly susceptible and moderately susceptible turfgrass.

**FIG 2:**
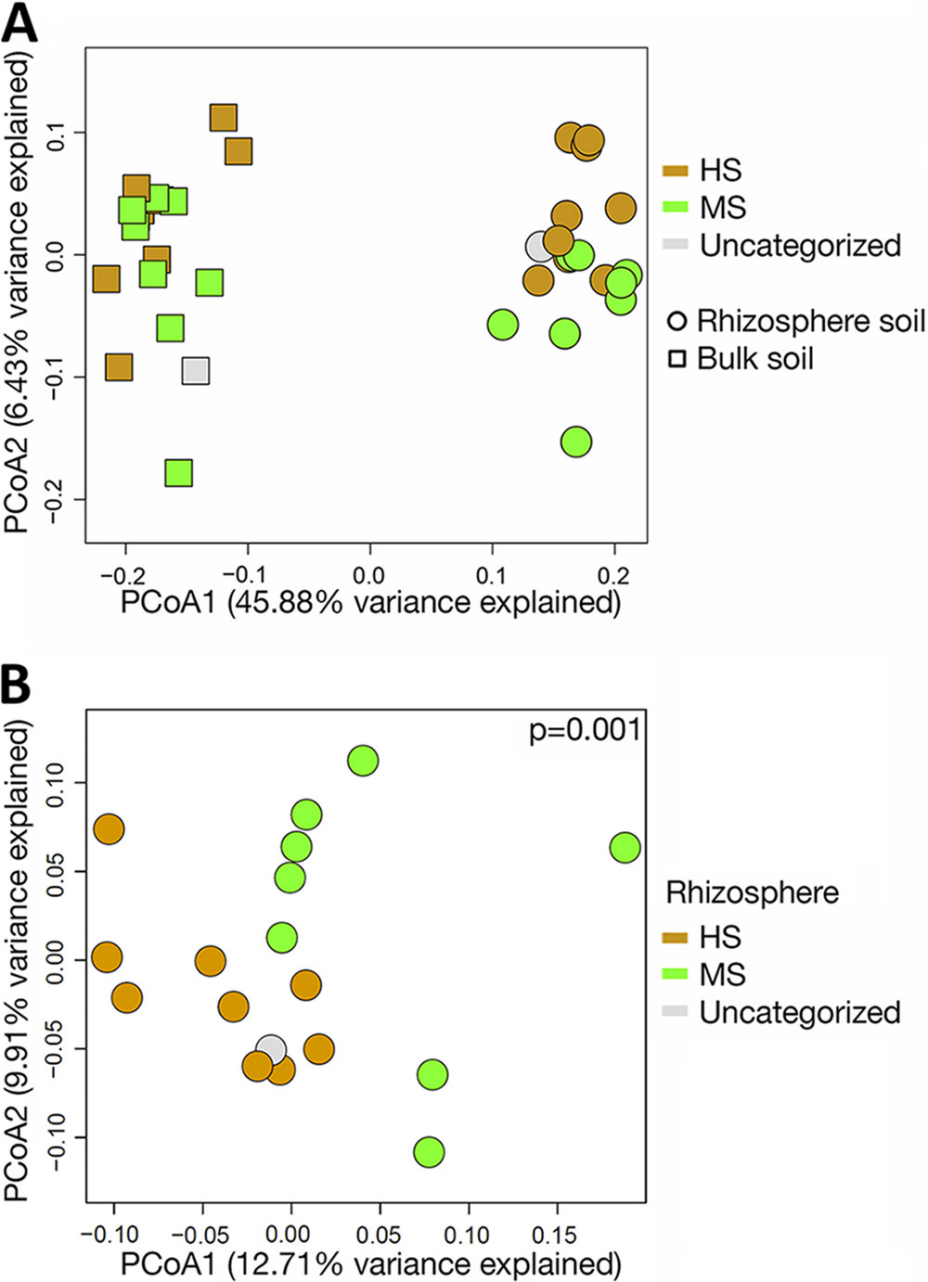
Principal-coordinate analysis (PCoA) of bulk soil versus rhizosphere microbiome (A) and MS versus HS turfgrass rhizosphere microbiome (B). Significant differences between MS and HS samples were tested using PERMANOVA. MS and HS are disease susceptibility groups derived from the peak disease development stage.

**FIG 3:**
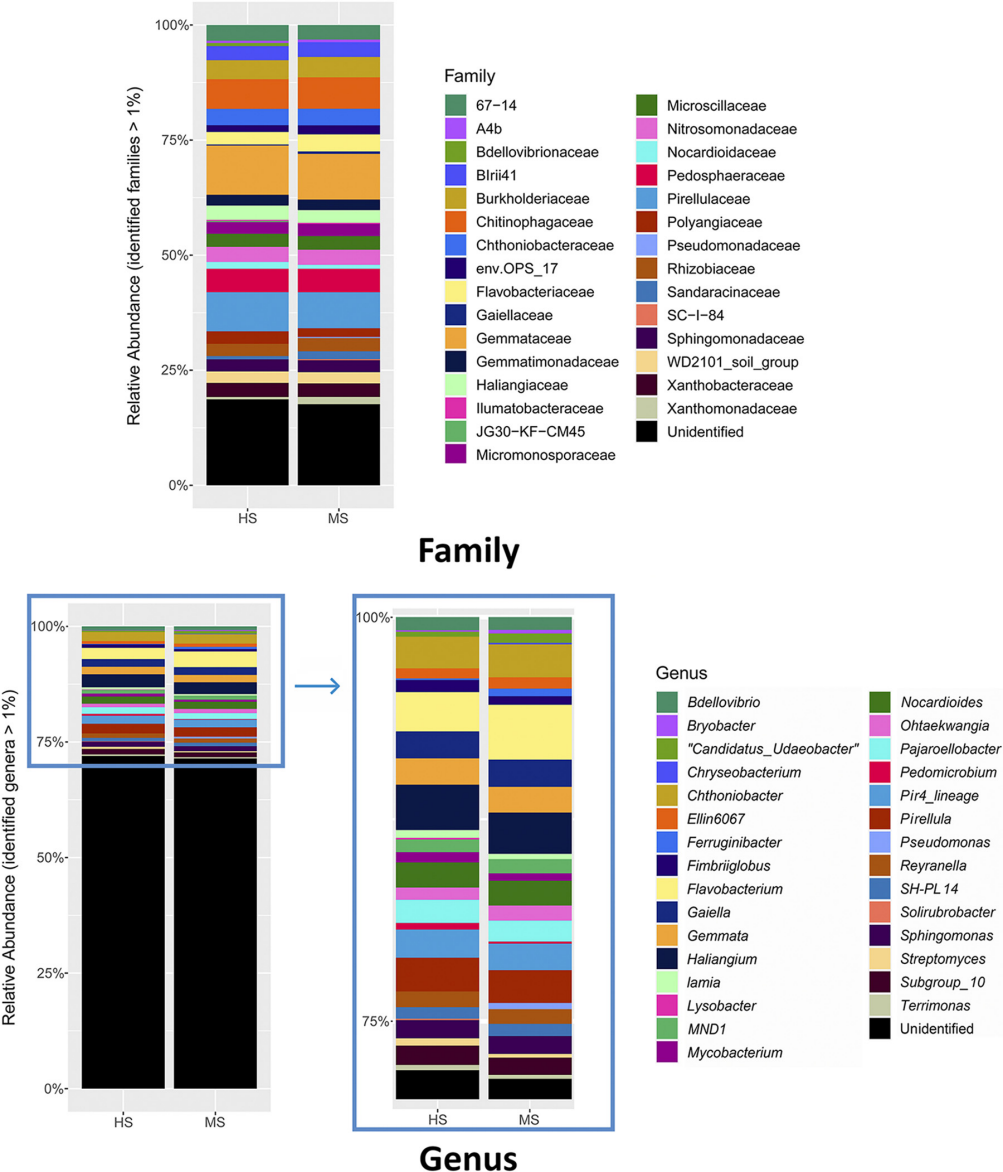
Average relative abundances of rhizosphere microbiome from MS and HS turfgrass for the taxa that represent more than 1% of the identified community at family (top) and genus (bottom) levels. MS and HS are disease susceptibility groups derived from the peak disease development stage.

**FIG 4:**
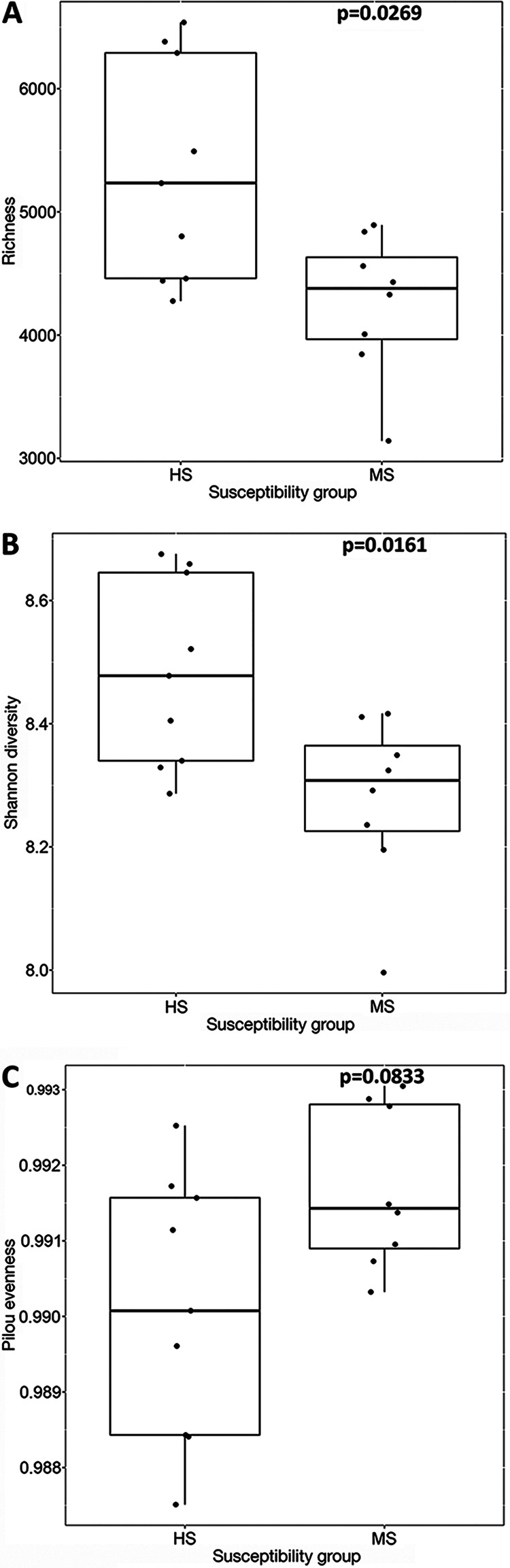
Bacterial richness (A), Shannon diversity (B), and Pilou evenness (C) in rhizosphere of turfgrass from MS and HS susceptibility samples at peak disease development stage at the ASV level. The natural logarithms were used in Shannon index calculation. The significance tests was performed using a nonparametric Wilcoxon rank-sum test.

Two-dimensional principal-coordinate analysis (PCoA) showed that distinct bacterial community structures existed between the bulk and rhizosphere soil and between the rhizosphere soil of HS and MS samples (Fig. 2). PERMANOVA statistically confirmed the visual observations of bacterial community composition differences between sample types (Fig. 2A) and susceptibility groups of rhizosphere soil (Fig. 2B). Although the overall rhizosphere bacterial compositions were different between MS and HS turfgrass, the major microbial taxa were identical when analyzed at family and genus levels, with less than 20% and more than 75% of the taxa unidentified at each taxonomic level, respectively (Fig. 3). The dominant families identified included *Gemmataceae*, *Pirellulaceae*, *Chitinophagaceae*, *Pedospheraceae*, and *Burkholderiaceae*; and the dominant genera identified included *Flavobacterium*, *Haliangium*, *Chthoniobacter*, *Pirellula*, and *Gaiella* (Fig. 3). The majority of the rhizosphere soil amplicon sequence variants (ASVs) are shared between the HS and MS turfgrass (8,077), with more ASVs being unique to HS (1,181) than MS (347). Highly susceptible turfgrass samples also had a higher microbial α-diversity relative to samples from MS turfgrass, as shown by richness (Fig. 4A), Shannon index (Fig. 4B), and Pilou evenness (Fig. 4C).

**FIG 5:**
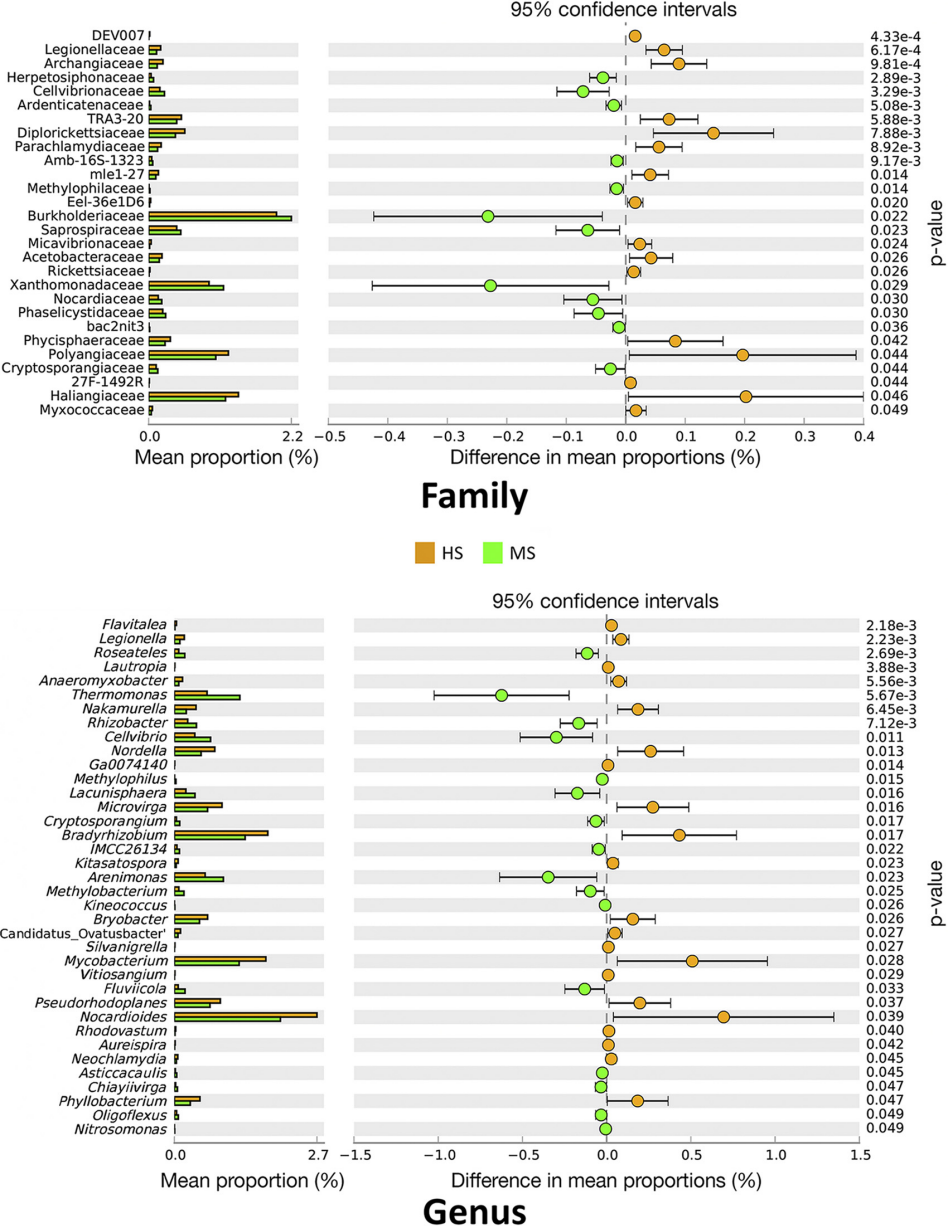
Rhizosphere bacterial taxa relative abundance differences between the HS and MS turfgrass at family (top) and genus (bottom) level tested with Welch’s *t* test. MS and HS are disease susceptibility groups derived from the peak disease development stage.

**FIG 6:**
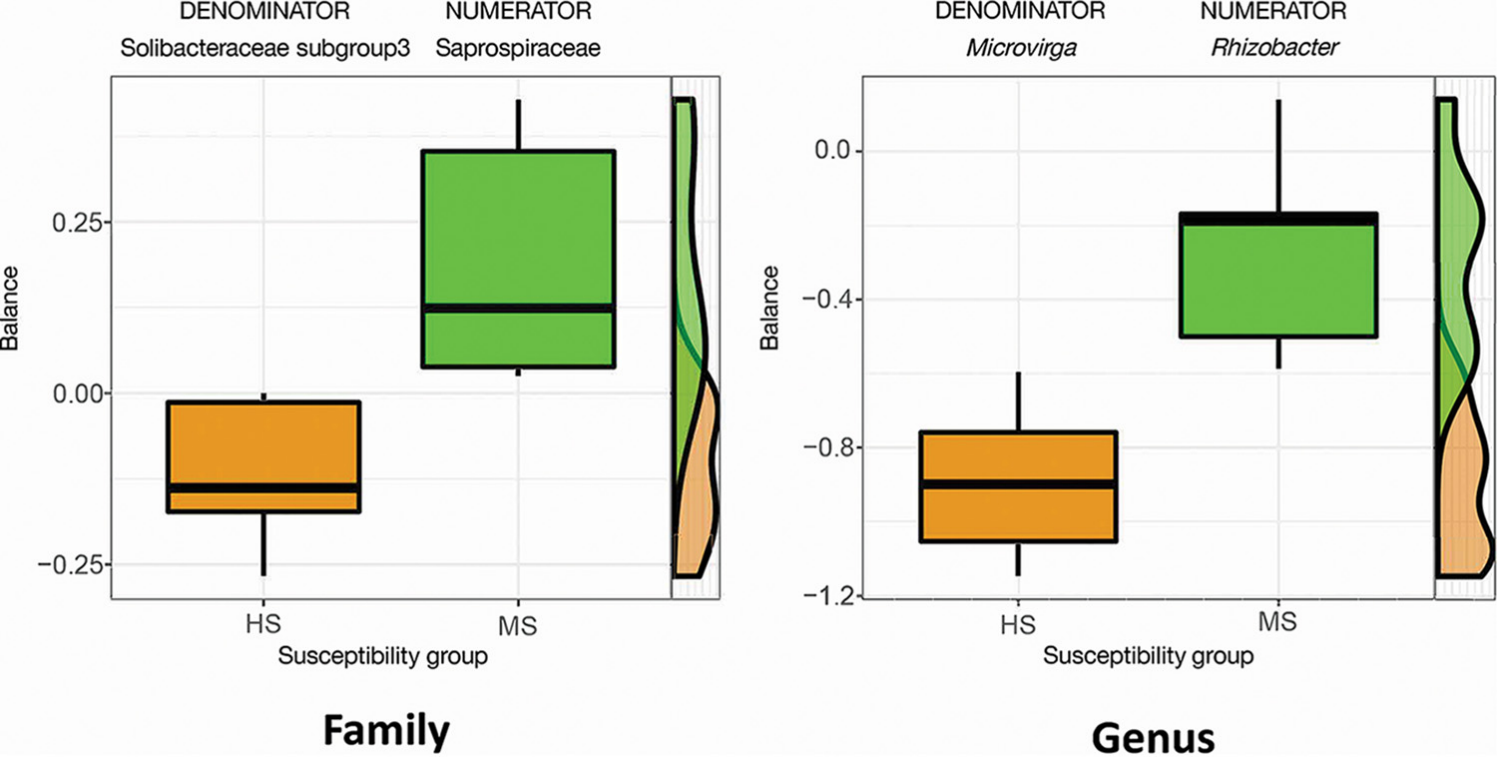
Compositional balance change analysis identifying the bacterial signatures that discriminate the rhizosphere microbiome between HS and MS turfgrass. The balance indicates the logarithm ratio of the relative abundance of identified denominator and numerator. MS and HS are disease susceptibility groups derived from the peak disease development stage.

In the rhizosphere, there were 28 families and 32 genera that differed in relative abundance between HS and MS samples according to Welch’s *t* test without false discovery rate (FDR) correction (Fig. 5). When the FDR correction was applied, no differences in relative abundance between HS and MS samples were detected at the family and genus levels. A balance analysis that accounted for the compositional nature of the data set was also performed to detect the microbial signature for discerning the HS and MS rhizosphere bacterial community. The signatures were determined by searching the association between the factor for overall bacterial microbiome difference with the bacterial taxa balances defined as the normalized log ratio of the geometric mean of the numerator and denominator bacterial taxa. The results showed that relative abundance log ratios of *Rhizobacter* (numerator) to *Microvirga* (denominator) at the genus level and *Solibacteraceae* subgroup3 (numerator) to *Saprospiraceae* (denominator) at the family level were robust microbial signatures to differentiate the HS and MS turfgrass rhizosphere bacterial community, with an adjusted area under the receiver operating characteristic curve for cross-validation equal to 0.9875 and 0.983 for genus and family level, respectively (Fig. 6).

**FIG 7:**
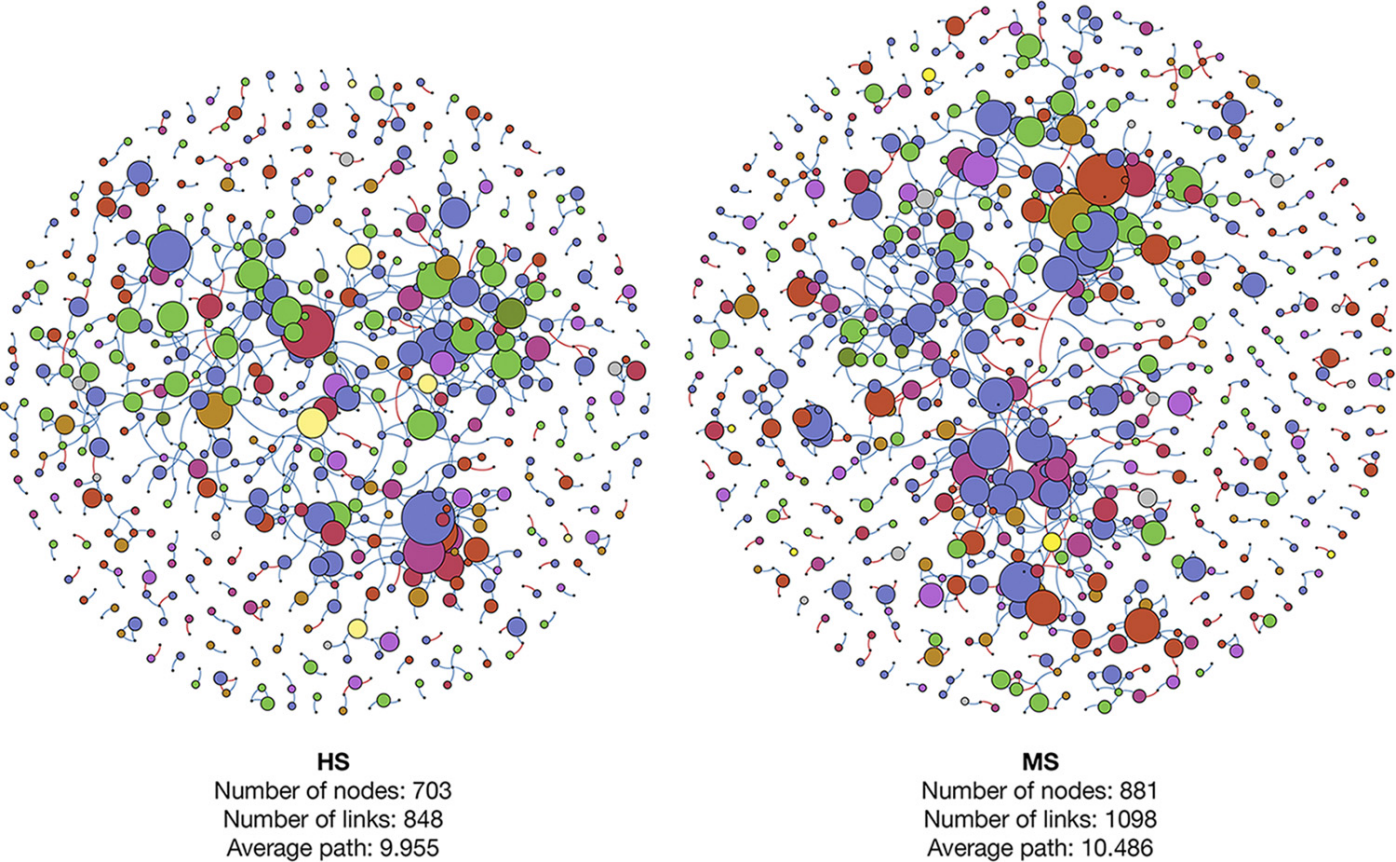
Rhizosphere soil bacterial microbiome co-occurrence networks at phylum level of MS and HS turfgrass, in which the sizes of the nodes were scaled based on in-degree values; blue and pink paths represent positive and negative correlations, respectively.

**FIG 8:**
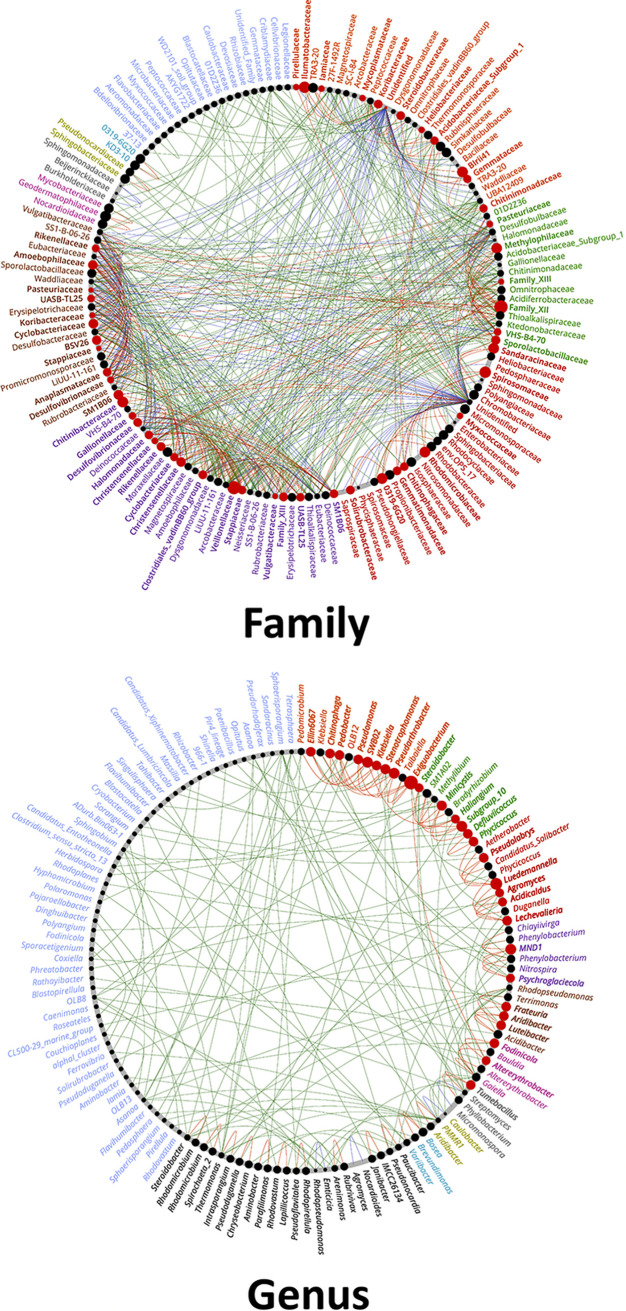
NetShift analysis by comparing the co-occurrence networks between the MS and HS turfgrass to identify the driver taxa at family (top) and genus (bottom) levels, where the nodes were scaled based on the degree in neighbor shift. The red nodes are the identified important drivers responsible for the network shift between the MS and HS turfgrass rhizosphere microbiome; and the green, red, and blue paths represent the edges shown in MS, HS, and both, respectively. The node label color scheme represents the network module. MS and HS are disease susceptibility groups derived from the peak disease development stage.

A co-occurrence network analysis was performed to visualize the microbial interaction of HS and MS turf rhizosphere soil bacteria and showed different network patterns (Fig. 7). The HS co-occurrence network had fewer nodes, internode links, average path, and more *Actinobacteria* and *Firmicutes* involved in the major modulations than that of the MS network. The co-occurrence networks were then further analyzed using “NetShift” to quantify the differences and identify the keystone microbial taxa that triggered the shift of the microbial networking between HS and MS rhizosphere bacterial communities when clustered at the family and genus level (Fig. 8). There were 55 families and 28 genera identified as driver taxa when comparing HS and MS co-occurrence networks aggregated at each taxonomic level.

**FIG 9:**
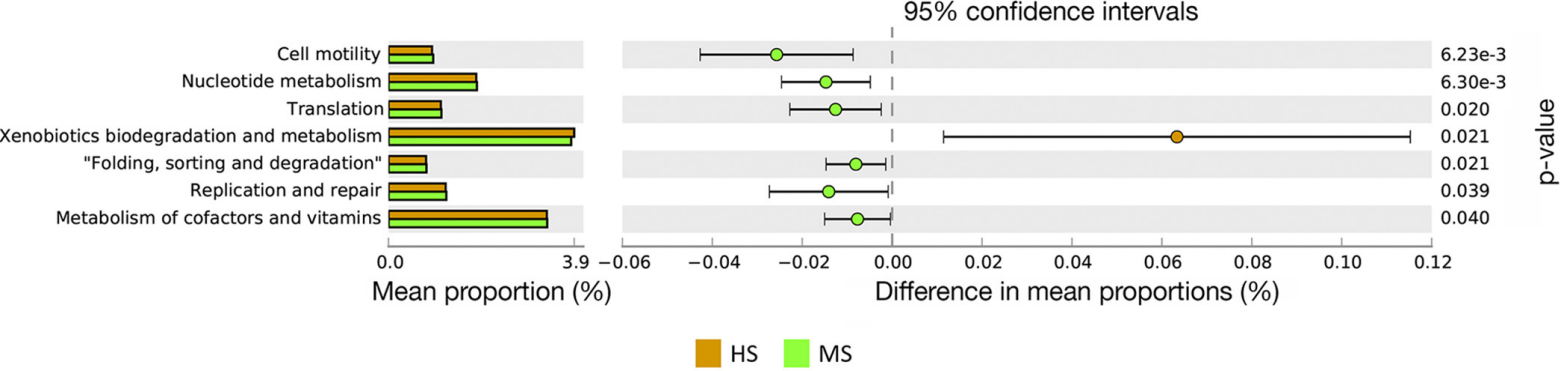
Significant differences in predicted rhizosphere microbiome functional pathways of MS and HS turfgrass using Tax4Fun2 and tested by Welch’s *t* test. MS and HS are disease susceptibility groups derived from the peak disease development stage.

Rhizosphere soil bacterial function was predicted using Tax4Fun2 (24) to explore the potential microbial functional differences between HS and MS samples during the peak disease development period. Predicted functional pathways at level two according to KEGG reference for molecular functions of genes (25), including nucleotide metabolism, folding, sorting and degradation, cell motility, translation, transcription, replication and repair, and metabolism of cofactors and vitamin-associated genes, were found to be more abundant in the rhizosphere of MS samples (Fig. 9). In the HS samples, rhizosphere genes associated with xenobiotic biodegradation and metabolism pathways were more abundant (Fig. 9). These comparisons were performed using Welch’s *t* test, and no significant differences were detected when applying the FDR correction.

### Bulk soil nutrient and chemical property analysis.

Bulk soil chemical properties were compared among the three dollar spot severity groups throughout the incubation period. The bulk soil was sampled prior to the inoculation of *C. jacksonii* to evaluate if bulk soil chemical properties explained the turfgrass responses to the pathogen inoculation. The results showed that iron concentration was significantly lower (*P* = 0.005) in the high disease group (0.81 mg iron/kg soil) than in the low disease group (0.95 mg/kg) throughout the peak disease development stage from 4 to 10 DAI (Table 2 and 3) according to Kruskal-Wallis test and followed by Steel-Dwass paired comparison. Bulk soil iron was also lower in the HS samples than in the MS samples (*P* = 0.0021) according to Kruskal-Wallis test following recategorization of the samples (see Table S1 in the supplemental material). A Mantel test was conducted to determine the correlation between the overall soil chemical properties and the soil bacterial community (Table 4). Bulk soil chemical properties did not correlate with the bulk soil bacterial community (r = −0.2297, *P* = 0.966), but they did correlate with the rhizosphere bacterial community (*r* = 0.274, *P* = 0.048) (Table 4). To further examine the relationship between bulk soil chemical properties and dollar spot severity during the peak disease development stage, a backward stepwise regression model was constructed after removing significant colinear variables. The stepwise model (adjusted r^2^ = 0.5041, *P* = 0.002031) suggested that soil iron significantly (*P* = 0.00062) and positively regressed with average turfgrass greenness during the peak development period (Table 5).

**TABLE 2:** Mean separation of turf-associated bulk soil chemical elements^*a*^

Element or parameter	DAI 0		DAI 2		DAI 4		DAI 6		DAI 8		DAI 10		DAI 12		DAI 14		DAI 16	
	ChiSq	*P* value	ChiSq	*P* value	ChiSq	*P* value	ChiSq	*P* value	ChiSq	*P* value	ChiSq	*P* value	ChiSq	*P* value	ChiSq	*P* value	ChiSq	*P* value
pH	0.626	0.060	4.526	0.104	3.170	0.205	1.509	0.470	1.064	0.587	2.561	0.278	2.012	0.366	2.667	0.264	2.667	0.264
OM	0.986	0.611	0.184	0.912	5.535	0.063	2.611	0.271	0.784	0.676	1.092	0.579	0.246	0.884	0.012	0.994	0.012	0.994
Al	3.942	0.139	6.398	0.041*	8.082	0.018*	5.661	0.059	4.012	0.135	9.310	0.01**	3.310	0.191	3.193	0.203	0.319	0.203
Ca	1.977	0.372	2.854	0.240	5.719	0.057	9.310	0.01**	2.561	0.278	5.485	0.064	1.450	0.484	0.889	0.641	0.889	0.641
Cu	2.117	0.347	10.714	0.005**	4.667	0.097	2.328	0.312	0.363	0.834	2.538	0.281	3.170	0.205	0.924	0.630	0.924	0.630
Fe	5.099	0.078	3.509	0.173	9.275	0.01**	7.906	0.019*	8.924	0.012*	11.614	0.003**	4.714	0.095	4.994	0.082	4.994	0.082
K	0.363	0.834	0.152	0.927	3.521	0.172	7.029	0.030	3.193	0.203	2.047	0.359	0.328	0.849	1.263	0.532	1.263	0.532
Mg	1.063	0.587	2.538	0.281	3.661	0.160	4.678	0.096	0.854	0.653	3.170	0.205	1.275	0.529	0.667	0.717	0.667	0.717
Mn	3.029	0.220	0.877	0.645	1.205	0.548	1.509	0.470	2.632	0.268	3.895	0.143	0.503	0.778	0.246	0.884	0.246	0.884
Mo	0.456	0.796	0.222	0.895	0.714	0.700	5.556	0.062	1.556	0.459	0.714	0.700	0.105	0.949	0.737	0.692	0.737	0.692
Na	1.310	0.520	0.152	0.927	4.012	0.135	3.825	0.148	0.667	0.717	3.790	0.150	0.737	0.692	0.421	0.810	0.421	0.810
P	3.240	0.198	1.368	0.505	1.298	0.523	2.246	0.325	0.877	0.645	1.064	0.587	0.714	0.700	0.573	0.751	0.531	0.751
S	1.556	0.459	0.105	0.949	0.140	0.932	0.433	0.805	0.561	0.755	0.035	0.983	2.117	0.347	2.117	0.347	2.117	0.347
Zn	1.298	0.523	3.415	0.181	0.012	0.994	0.246	0.884	1.509	0.470	1.064	0.587	0.246	0.884	1.275	0.529	1.275	0.529
C	1.064	0.587	3.193	0.203	0.667	0.717	0.246	0.884	1.450	0.484	0.012	0.994	3.193	0.203	2.538	0.281	2.538	0.281
N	1.485	0.476	2.819	0.244	0.152	0.927	1.766	0.414	1.205	0.548	0.152	0.927	3.614	0.164	2.538	0.281	2.538	0.281

a Nonparametric Kruskal-Wallis tests conducted to test the significance level. Asterisks indicate the significance level; *, *P* < 0.05 and **, *P* < 0.01. DAI, days after inoculation of *C. jacksonii*; ChiSq, chi-square.

**TABLE 3:** Bulk soil iron content of each severity group categorized based on the peak of disease development stage

Group	Iron content^*a*^ (mg/kg of dry soil) at:			
	DAI 4	DAI 6	DAI 8	DAI 10
High	0.825 ± 0.035b	0.811 ± 0.037b	0.818 ± 0.035b	0.795 ± 0.031b
Low	0.989 ± 0.035a	0.975 ± 0.037a	0.987 ± 0.035a	0.989 ± 0.031a
Medium	0.859 ± 0.035b	0.887 ± 0.037ab	0.868 ± 0.035ab	0.889 ± 0.031ab

a All values are mean ± SE. Different lowercase letters indicate significant differences derived from Steel-Dwass paired comparisons with an α value of 0.05.

**TABLE 4:** Correlations among bulk soil chemical property, bulk soil microbiome, and rhizosphere microbiome^*a*^

Correlation	Mantel statistic	
	*r*	*P* value
Soil chem vs bulk microbiome	−0.230	0.97
Soil chem vs rhizo microbiome	0.245	0.048*
Bulk microbiome vs rhizo microbiome	−0.065	0.58

a Performed using Mantel tests. An asterisk indicates the significance level; *, *P* < 0.05.

**TABLE 5:** Stepwise regression results^*a*^

Coefficient	Estimate	SE	t-value	P(>|t|)
Intercept	27.09	13.06	2.075	0.056
Fe	69.74	16.19	4.309	<0.001***
Zn	−27.28	15.81	15.81	0.105

a Selection of the optimal regression model for bulk soil chemical elements and average dollar spot disease severity (greenness) during the peak disease development stage (4 to 10 DAI). Asterisks indicate the significance level: ***, *P* < 0.001. Residual standard error, 6.011 on 15 degrees of freedom; multiple *r*^2^, 0.5624; F-statistic: 9.641 on 2 and 15 DF, *P* = 0.002031.

## DISCUSSION

The results from this study indicated that initial differences in the soil rhizosphere bacterial community can impact the level of dollar spot development in the turfgrass canopy. These differences occurred over small areas despite uniform host plants and seemingly uniform environmental conditions. The mechanisms of disease suppression provided by the rhizosphere community were not directly studied, and the short-amplicon sequencing method used in the study yielded only compositional data. As such, these results provide limited resolution for exploring the specific species and genera, but differential analysis of microbial taxa relative abundances and NetShift analysis of co-occurrence networks in this study provide supporting information for the hypothesis that disease suppression is related to the occurrence of antagonistic organisms in the rhizosphere. A similar hypothesis was also suggested in work done by Wei et al. (22), which indicated that the rhizosphere bacterial community determined the disease occurrence and severity caused by Ralstonia solanacearum in tomato plants and specifically linked disease suppression to the antagonistic activity of soil bacteria in the genera *Bacillus* and *Pseudomonas*.

In our study, differential analysis revealed that certain families and genera were higher in relative abundance in the rhizosphere of MS samples than those in HS samples. These families, including *Nocardiaceae* and *Xanthomonadaceae*, and genera, including *Rhodococcus* and *Janthinobacterium*, are known to produce a range of antimicrobial compounds (26–29). Among the microbial co-occurrence network shift drivers identified through NetShift, node betweenness was significantly increased in MS samples for certain genera, including *Pseudonocardia*, *Streptomyces*, and “*Candidatus* Entotheonella,” which are all known for their ability to produce antifungal compounds (30–32). The betweenness represents the number of paths that pass through a node in which the paths are part of the shortest path of any two given nodes. Simply, the increase in node betweenness for these genera indicated that more co-occurrent microbes are influenced by these genera, making them important in the overall community and offering a possible explanations for microbial suppression of dollar spot in MS turf samples.

The predicted microbial function and relative abundance differences in this study were detected using Welch’s *t* test without FDR correction. When the FDR correction was applied, however, no significant differences in relative abundance were observed. The FDR correction is used to reduce the possibility of false-positive results when conducting multiple comparisons, with the embedded trade-off false-negative results even for the less stringent Storey FDR correction. As the number of hypothesis tests increase, the resulting adjusted *P* value will become more conservative. This study conducted approximately 1.25 × 10^5^ hypothesis tests (paired comparisons for 468 families and 533 genera) for each taxonomic level using normalized data to overcome the read inequality among the samples, which may be overly conservative in identifying differences when an FDR correction is applied. An important assumption of using the FDR is that the multiple hypothesis tests are independent or weakly dependent of one another. However, as multiple influential taxa exist in the examined communities in this study, the assumption of applying FDR is not necessarily appropriately met. This study aimed to provide initial explorations into the potential microbial factors that trigger hypervariable disease severity over small distances and determine avenues for future, more rigorous mechanistic research. We have provided the results of both the *t* test and FDR analyses here so that future research can build off these results but so that proper context into the potential significance of the results can also be provided.

How the microbes found in the soil can be antagonistic to a foliar pathogen like *C. jacksonii* remains unclear. Previous studies have suggested possible mechanisms via endophytic recruitment of functional rhizosphere microbes (33, 34) and direct uptake of antibiotic metabolites produced by soil microbes (35, 36). The effect of antibiotic uptake by the plants may also be further amplified through bioconcentration, especially for small molecules such as oxytetracycline and sulfonamides (37). In addition, the close spatial distance between the turfgrass canopy and the soil may also allow the soil microbial activity to have an impact on dollar spot expansion horizontally given that *C. jacksonii* relies on hyphae to extend the infection zone from one plant to another.

A number of other bacterial taxa with environmental or plant functional importance in the rhizosphere differed between the HS and MS samples. The balance analysis revealed that the log ratios of *Saprospiraceae* and *Solibacteraceae* subgroup3 at the family level and *Rhizobacter* to *Microvirga* at the genus level can effectively differentiate between the rhizosphere bacterial microbiomes of the HS and MS groups. Among these identified signatures, the family *Saprospiraceae* and genera *Rhizobacter* and *Microvirga* were also found to differ in relative abundances. Microbial species under the genus *Microvirga* include many root symbionts (38), whereas members of the *Rhizobacter* genus are common rhizobacteria (39), and they can also be plant pathogenic (40). Although the relative abundances were low, these identified taxa served as key signatures to differentiate the HS and MS rhizosphere bacterial community and may also have functional importance. For example, the identified family signature *Saprospiraceae* was present at a low level in our study (<1% in relative abundance), but members of the *Saprospiraceae* family are known to break down complex organic compounds in the environment (41) and are also suggested to have functional importance while underrepresented in soil abundance (42). The manner in which these microbial signatures interacted with the pathogen and host plant and whether they can be used for future evaluations of dollar spot suppression require further research.

Functional prediction was performed to better understand the implications of the differences identified in bacterial community composition and interaction of HS and MS samples in the absence of a comprehensive metagenomic analysis. The MS rhizosphere bacterial community was more enriched in genetic information processing and cellular processes metabolic pathways, whereas the HS rhizosphere bacterial community was more abundant in predicted xenobiotic biodegradation and metabolism. This result could help explain why the HS rhizosphere bacterial community resulted in more susceptible turfgrass. Many chemical compounds, such as salicylic acid (SA) analogs and β-aminobutyric acid, can induce plant systemic acquired resistance that primes plants to defend against pathogens through activation of SA or abscisic acid (ABA) signaling pathways (43). The higher predicted relative abundance in genes associated with xenobiotic biodegradation and metabolic pathways in the HS rhizosphere bacterial community suggested that the bacterial community has a higher potential to degrade xenobiotics, such as agrochemicals, transformation products, and secondary metabolites, that either has direct antagonistic effects on pathogen growth or compounds that have roles in priming plants against pathogens. These differences in relative abundances were relatively minor and without FDR correction and require further investigation using quantitative PCR (qPCR) or metagenomic tools for a more complete understanding.

In the study by Wei et al. (22), structural and functional differences in the rhizosphere bacterial microbiome were found to be the sole factors determining disease severity on tomato. In our study, bulk soil iron concentration predicted the disease susceptibility as well as that of the rhizosphere bacterial microbiome and seemed to contribute significantly to dollar spot suppression. Gu et al. (44) recently showed that siderophore production as a result of bacterial competition for iron resources in the soil environment strongly mediates R. solanacearum activity in the tomato rhizosphere. Specifically, iron-scavenging siderophores produced by nonpathogenic members of the bacterial consortia enhanced the fitness of these nonpathogenic bacteria in the soil environment and suppressed pathogen growth. Further large-scale screening of all major bacterial phylogenetic lineages established a strong positive linkage between inhibitory siderophore production by nonpathogenic bacteria and R. solanacearum suppression, indicating that the relative abundance of bacteria that produce pathogen-unusable siderophores in the tomato rhizosphere microbiome served as an effective predictor for disease outcome (45). These studies were done in a soilborne pathosystem, and it is unclear how pathogen-suppressing siderophore producers in the rhizosphere would compete with *C. jacksonii*, which is a foliar pathogen and possible saprophyte in the thatch layer. Other mechanisms are likely involved, such as iron directly or indirectly neutralizing pathogen activity. For example, Gadd (46) observed that oxalic acid, a potential virulence factor produced by *C. jacksonii* and several other important plant pathogens, can react with the free iron in the plant-soil interface and precipitate as crystalline or amorphous solids. Also, in iron-deficient soils, induced bacterial production of the siderophore pyoverdine repressed the expression of plant defense-related genes, such as the genes involved in SA and ABA pathways, which can lead to a higher plant susceptibility to diseases (47).

Low soil iron can also lead to low iron in the plant tissue. Iron plays multifaceted roles in plant defense mechanisms and plant-pathogen interactions (48). For example, iron serves as a key factor in plant disease defense via numerous regulatory genes involved in microbe response and plant homeostasis, including upregulating the transcription of pathogenesis-related genes and catalyzing the reactive oxygen species when attacked by pathogens (49, 50). Unbalanced iron homeostasis in plants can have serious impacts on disease outcomes. Low iron in Arabidopsis thaliana led to more severe Dickeya dadantii infection due to less ferritin coding transcript AtFER1, callose deposition, and reactive oxygen species production (51). These collective studies on low soil and plant iron may help explain how samples with lower soil iron in our study can lead to higher dollar spot susceptibility in turf and vice versa, especially under the high pH conditions (average pH, 7.241) present in our study (52).

Numerous field and *in vitro* studies have shown the beneficial effect of iron in plant disease suppression (53–55). The beneficial effects of iron are often found in conjunction with a pathogen-suppressive soil microbiome (17, 23), even though some studies have also shown an adverse effect of iron in Fusarium wilt (Fusarium oxysporum) disease on flax (Linum usitatissimum) and banana (*Musa* spp.) (56, 57). Healthy blueberry (Vaccinium corymbosum) plants were found to be associated with more diverse rhizosphere bacterial communities and higher iron content in the roots than unhealthy plants (23). An *in vitro* study demonstrated that soil Fe-ethylenediamine-N,N′-bis(2-hydroxyphenylacetic acid) (EDDHA) amendment has an additive and complementary effect in suppressing Fusarium wilt (Fusarium oxysporum f. sp. *cubense*) disease severity in banana (*Musa* spp.) grown in a disease-suppressive soil (17). The mechanisms of such a complementary effect of iron in our study remain unclear, but the Mantel test results suggest that the rhizosphere bacterial community was likely mediated by an interaction between soil iron levels and turfgrass plants, which in turn impacted disease development.

The rhizosphere microbiome is recruited or expelled from the bulk soil through the production of phytochemicals (58, 59), including many organic acids and secondary metabolites (60). More specifically, previous work by Pii et al. (61) demonstrated that plant iron status had a significant impact on the formation of rhizosphere microbiome structures, possibly via the release of different qualitative and quantitative root exudates. In our study, higher Fe in the bulk soil of MS samples may have induced production of root exudates that then recruited a particular rhizosphere bacterial community that was more suppressive to dollar spot development.

This study revealed several factors that may contribute to the hyperlocal variation in dollar spot disease development commonly observed in amenity turfgrass. Our findings suggest that putative antibiotic-producing members in the rhizosphere bacterial community play a role in the suppression of dollar spot on turfgrass. Furthermore, soil iron-plant interactions regulated the assembly of a suppressive rhizosphere microbiome, and this soil-plant-microbe interaction ultimately resulted in the observed variation in disease development on monocultured turfgrass over a small area. Future studies on whether the disease-suppressive function can be transplanted into a nonsuppressive soil, and how turfgrass physiologically mediates root exudates to recruit a disease suppressive rhizosphere microbiome by responding to different levels of soil iron, will be critical in further exploring the hypotheses raised by this research. Measuring enzyme activities using assays such as fluorescein diacetate hydrolysis will provide insights into the actual microbial functions. In addition, study of other communities in the microbiome, such as archaea, fungi, and protozoa, will yield a more comprehensive understanding of the plant-soil-microbe interactions and may provide novel strategies for disease suppression.

## MATERIALS AND METHODS

### Experimental design, sampling scheme, and sample preparation.

The experiment was conducted on a mature stand of creeping bentgrass (Agrostis stolonifera ‘Alpha’) at the O. J. Noer Turfgrass Research Facility in Verona, WI. The turf was grown on a native Troxel silt loam and mowed three times per week at a height of 1.25 cm. Eighteen turfgrass samples and the associated soil were randomly taken using a soil sampler with a 13-cm diameter and a 15-cm depth in a 256-m^2^ square plot on 10 October 2019. The samples were divided into a top layer (the top 7.5 cm) and a bottom layer (7.5- to 15-cm depth) by carefully inserting the soil sampler to the specified depths. Due to the nature of the turfgrass and soil properties, there was hardly any soil without direct contact with roots in the top layer and rarely any root presence in the bottom layer. Therefore, we defined the bulk soil as the soil from the bottom layer without direct root contact. The soil samples of each layer were stored separately as turf and bulk soil samples. The turf samples were then used for inoculation experiments after they were subsampled for rhizosphere microbiome analysis. Bulk soil samples were subsampled from the homogenized bottom layer soil for both microbiome and chemical property analysis. Two 1-cm-diameter subsamples to 5-cm depth containing approximately 10 to 15 individual creeping bentgrass plants were taken from each turf sample for microbiome analysis using a custom-made soil probe, which was converted from a golf club that produced soil cores in a 1-cm-diameter circular shape. The subsamples from the same turf sample were immediately crushed with a sterile spatula and tweezer, and the soil loosely attached to the root system was separated from plant and rhizosphere soil by aggressively shaking into a sterile glass petri dish. Rhizosphere soil that remained closely attached to the root was then carefully collected using a spatula by avoiding the root tissues. The intact turf samples, from which the subsamples were taken, were then inoculated with 1 milliliter of dollar spot inoculum using a vaporizer within 1 h of sampling. The dollar spot inoculum was created by growing *C. jacksonii* in potato dextrose broth for 72 h, rinsing three times in distilled water, and homogenizing in sterile 0.85% saline water in a blender for 1 minute. The final inoculum had an approximate *C. jacksonii* density of 4.1 × 10^4^ CFU/ml, as determined by testing with triplicated serial dilutions on potato dextrose agar.

After inoculation, the turf samples were incubated in a growth chamber at 25°C, 70% relative humidity, and 15-h photoperiod. Each sample was placed on a sterile filter paper with an individual glass water pan. The turf samples were maintained at a 0.5-cm height using sterile scissors, supplied with distilled water through wetting the filter paper, and measured for dollar spot severity every other day for 16 days (see Fig. S1 in the supplemental material). Dollar spot severity was assessed by taking digital photos 30 cm directly above the turf surface and counting the percentage of green pixels using imageJ. Bulk soil samples were sent to the Cornell Nutrient Analysis Laboratory (Ithaca, NY) to analyze the chemical properties, including pH; organic matter content; and Al, Ca, Cu, Fe, K, Mg, Mn, Mo, Na, P, S, Zn, C, and N content according to procedures outline in Gugino et al. (62). Briefly, soil samples were dried in open containers overnight and sieved to remove pebbles and plant tissues. Soil organic matter content was measured by dry combustion at 550°C for 2 hours, and pH was measured as 1:1 soil to water solution by volume using an automatic pH probe (Lignin, Albuquerque, NM). Soil nutrients were extracted using Morgan’s solution and quantified with inductively coupled argon plasma spectrophotometry (Thermo Fisher Scientific, Cambridge, UK).

### Library preparation and short-amplicon sequencing.

Overall, 36 soil samples (18 bulk soil and 18 rhizosphere soil samples) along with 1 negative control containing PCR-grade water were used in this study. For each sample, 0.25-g soil was used for DNA extraction using a DNeasy PowerLyzer PowerSoil kit (Qiagen Inc., Germantown, MD) following the manufacturer’s protocol. All extractions were quantified for nucleic acid concentration using a NanoDrop 1000 instrument (Thermo Fischer Scientific, Waltham, MA). The PCR was performed according to Dill-McFarland et al. (63) with minor modifications. Briefly, each reaction contained 5 μl of the DNA template at 10 ng/μl, 12.5 μl Kapa HiFi HotStart ReadyMix, 6.5 μl PCR-grade water, and 0.5 μl of each barcoded forward and reverse primer (64), which targeted the v4 region of the 16S rRNA gene. The thermocycling conditions were 3 min at 95°C prior to 25 cycles of 30 s at 95°C, 30 s at 55°C, and 30 s at 72°C, with a final step of 5 min at 72°C. The amplicons were purified using a ZR-96 Zymoclean gel DNA recovery kit (Zymo Research, Irvine, CA) and normalized with a Mag-Bind EquiPure library normalization kit (Omega Bio-Tek Inc, Norcross, GA). The amplicons were then pooled and quantified to 4 nM with a Qubit double-stranded DNA (dsDNA) high-sensitivity (HS) assay kit (Thermo Fischer Scientific). The final pool was sequenced on Illumina MiSeq instrument with a 2 × 250-bp paired-end (PE) reagent kit v2 (Illumina, Inc., San Diego, CA) in the Biotechnology Center at the University of Wisconsin–Madison.

### Data analysis.

The raw sequences were processed using the software package “DADA2” in R 3.6.0. Forward and reverse reads were quality filtered to 248 and 160 bases, respectively, according to average quality score, allowing a maximum of 2 errors each of the reverse and forward read; denoised by learning the error rate from pooled reads; and merged to a target length between 243 and 253 bases. The taxonomy levels associated with each amplicon sequence variant (ASV) were assigned according to the SILVA database (v.132) after removing the chimeras using *de novo* identification (“consensus” method in function “removeBimeraDenovo”) in each sample. The ASV and taxonomic tables were then exported as .txt files and analyzed using R packages “phyloseq” and “vegan.” The reads for each sample were normalized using variance stabilizing transformation with the “DeSeq2” package due to a relatively even read variation among the samples in the library (65). Microbial compositional differences and correlations were analyzed using Bray-Curtis dissimilarity [“vegdist()” in “vegan”] and visualized using two-dimensional PCoA. Multivariate analysis was performed using PERMANOVA [“adonis()” in “vegan”] or paired PERMANOVA with Bonferroni correction when the independent variable had more than two levels, with the Bray-Curtis dissimilarity of the sample bacterial community as the dependent variable, the disease susceptibility or disease severity of each DAI as the independent variable alone with DAI or soil type as covariate when necessary, and each analysis with 999 permutations. The samples among variable levels were checked with dispersion using “betadisper()” in “vegan,” which were all nonsignificant. Shannon diversities of HS and MS were compared using a nonparametric Wilcoxon rank-sum test in JMP Pro 14 (SAS Institute, Cary, NC).

The microbial co-occurrence networks of HS and MS samples were constructed using Molecular Ecological Network Analysis (MENA) (66), which uses a random matrix theory (RMT)-based method to predict the microbial interactions and capture the magnitude of the interactions. The nodes and the edge lists were then imported into Gephi 0.9.2 (67) for network visualization. Since approximately 90% of the ASVs had less than 0.02% of overall reads, postnormalization ASVs that represented less than 0.02% of the total reads were filtered out to make the result more readable. The community of the HS and MS microbial networks were compared to quantify the rewiring of the taxa in the networks by calculating the neighborhood shift and change of betweenness for the nodes using NetShift (68). Nodes with the highest degree change among these parameters are considered drivers. When analyzed at family and genus levels, the ASVs were aggregated at each taxonomic level to create the edge list. Microbial balance analysis was performed using the “selbal” package in R at family and genus levels using unnormalized ASV counts, as the compositional nature of the short-amplicon sequencing result and the uneven sequencing depths were both accounted for in the analysis (69). Differential relative abundances were analyzed using Welch’s *t* test at a significance level of α = 0.05 in using Statistical Analysis of Taxonomic and Functional Profiles (STAMP) (70).

Rhizosphere microbiome functional prediction was performed using the R-based tool Tax4Fun2 (24), which used the sequences of the ASV to perform a blast search against the SILVA (v.132) reference genome database and to create a metagenome profile. The genetic functions were then assigned by BLASTp against KEGG orthology (KO) (25) as a reference database. Differences in functional pathways at level two were statistically analyzed using Welch’s *t* test in STAMP. The associations of Bray-Curtis dissimilarity among bulk soil chemical properties, bulk soil microbiome, and rhizosphere microbiome samples were examined using Mantel tests in R.

Soil chemical properties among the disease groups were statistically analyzed with the nonparametric Wilcoxon rank-sum test in JMP Pro 14 (SAS Institute, Cary, NC), and regression with average disease severity of peak disease development stage (4 to 10 DAI) was performed using a stepwise selection for the optimal predictive model in R. Collinearity variable selection and removal were performed using the customized function vif_func (71) for calculating the variance inflation factor. The best model was constructed with backward selection using the function stepAIC under package “MASS.”

### Data availability.

All raw sequences generated from this study were deposited at the NCBI Sequence Read Archive and are publicly accessible under the BioProject number PRJNA642971.

## Supplementary Material

Supplemental file 1
