# Supplementary material for: Hyperlocal Variation in Soil Iron and the Rhizosphere Bacterial Community Determines Dollar Spot Development in Amenity Turfgrass
Source: Appl Environ Microbiol. 2021 Apr 27;87(10):e00149-21. doi: 10.1128/AEM.00149-21 (PMC8117751; doi:10.1128/AEM.00149-21)
Supplement: Supplemental file 1 [file AEM.00149-21-s0001.pdf]

Supplementary Table S1. Wilcoxon non-parametric comparison of soil chemical properties (mg/kg) between HS and MS associated bulk soil. MS and HS are disease susceptibility groups derived from the peak disease development stage.

Property	Level	Mean	Std. Dev.	Std. Error Mean	Lower 95%	Upper 95%	Wilcoxon p-value
pH	HS	7.240778	0.233394	0.0777981	7.061375	7.420181	0.0543
	MS	7.398875	0.07535	0.0266401	7.335881	7.461869	
OM	HS	2.985949	0.275381	0.0917936	2.774273	3.197625	0.7361
	MS	3.076462	0.103921	0.0367415	2.989582	3.163342	
Al	HS	2.774205	0.252402	0.0841339	2.580192	2.968218	0.0161*
	MS	3.162284	0.29524	0.104383	2.915458	3.409111	
Ca	HS	1639.915	80.10273	26.700911	1578.342	1701.487	0.0433*
	MS	1714.069	110.1071	38.928753	1622.017	1806.121	
Cu	HS	0.080371	0.032808	0.0109359	0.055153	0.105589	0.2482
	MS	0.092079	0.020072	0.0070966	0.075298	0.10886	
Fe	HS	0.818683	0.097546	0.0325154	0.743702	0.893664	0.0021**
	MS	0.975093	0.055358	0.0195718	0.928813	1.021373	
K	HS	155.3454	11.78698	3.9289926	146.2852	164.4057	0.1489
	MS	161.5223	8.360104	2.955743	154.5331	168.5115	
Mg	HS	493.3613	26.29071	8.7635684	473.1525	513.5702	0.2898
	MS	507.6362	38.32882	13.551285	475.5925	539.6799	
Mn	HS	2.186369	1.367373	0.4557909	1.135313	3.237424	0.0833
	MS	3.398221	1.248337	0.4413539	2.354585	4.441857	
Mo	HS	0.005837	0.001988	0.0006626	0.004309	0.007364	0.9233
	MS	0.005627	0.001894	0.0006696	0.004044	0.00721	
Na	HS	27.50916	2.103361	0.7011202	25.89237	29.12594	0.5006
	MS	28.36482	1.818714	0.6430124	26.84434	29.8853	
P	HS	16.69933	1.557548	0.5191827	15.50209	17.89657	0.4414
	MS	15.66392	2.09468	0.7405812	13.91272	17.41511	
S	HS	4.721017	0.446798	0.1489327	4.377577	5.064456	0.5006
	MS	4.889829	0.443375	0.1567568	4.519157	5.2605	
Zn	HS	0.825432	0.158275	0.0527584	0.703771	0.947093	0.8474
	MS	0.849113	0.034658	0.0122533	0.820139	0.878088	
C	HS	1.798	0.085772	0.0285905	1.73207	1.86393	0.9233
	MS	1.843875	0.214384	0.0757961	1.664646	2.023104	
N	HS	0.170222	0.011998	0.0039992	0.161	0.179445	0.8474
	MS	0.176125	0.021649	0.0076542	0.158026	0.194224	

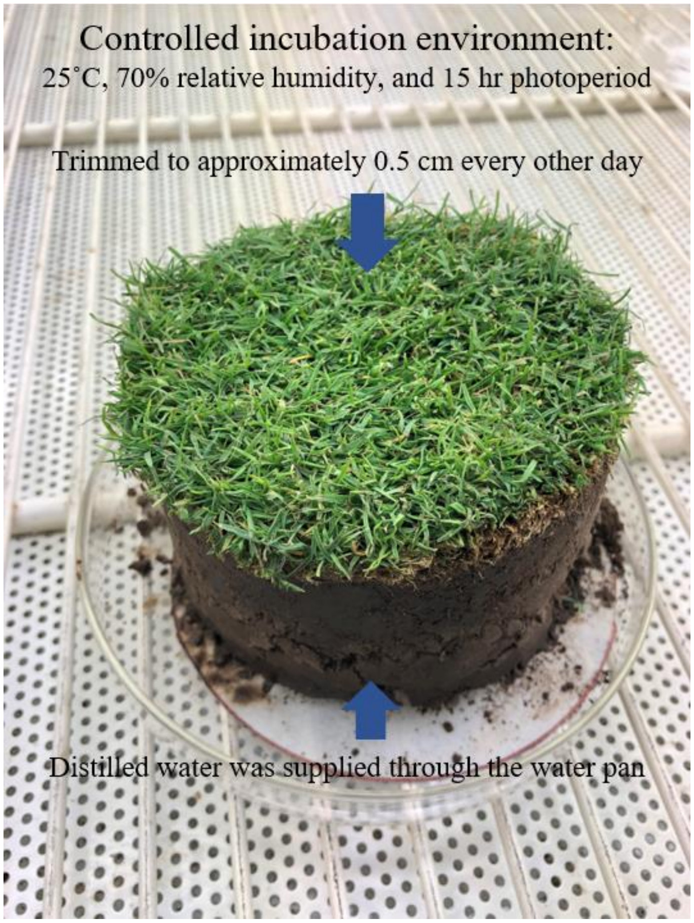

Supplementary Figure S1. Set-up of each turf sample in the controlled environment growth chamber for the incubation after inoculation with *C. jacksonii*.
